# Real-time real-world analysis of seasonal influenza vaccine effectiveness: method development and assessment of a population-based cohort in Stockholm County, Sweden, seasons 2011/12 to 2014/15

**DOI:** 10.2807/1560-7917.ES.2016.21.43.30381

**Published:** 2016-10-27

**Authors:** Amy Leval, Maria Pia Hergens, Karin Persson, Åke Örtqvist

**Affiliations:** 1 Department of Communicable Disease Control and Prevention for Stockholm County, Stockholm, Sweden; 2 Infectious Disease Unit, Karolinska Institutet, Department of Medicine Solna, Solna, Sweden; 3 Janssen-Cilag, Solna, Sweden; 4 These authors contributed equally to this manuscript

**Keywords:** Influenza, seasonal, vaccine, effectiveness

## Abstract

Real-world estimates of seasonal influenza vaccine effectiveness (VE) are important for early detection of vaccine failure. We developed a method for evaluating real-time in-season vaccine effectiveness (IVE) and overall seasonal VE. In a retrospective, register-based, cohort study including all two million individuals in Stockholm County, Sweden, during the influenza seasons from 2011/12 to 2014/15, vaccination status was obtained from Stockholm’s vaccine register. Main outcomes were hospitalisation or primary care visits for influenza (International Classification of Disease (ICD)-10 codes J09-J11). VE was assessed using Cox multivariate stratified and non-stratified analyses adjusting for age, sex, socioeconomic status, comorbidities and previous influenza vaccinations. Stratified analyses showed moderate VE in prevention of influenza hospitalisations among chronically ill adults ≥ 65 years in two of four seasons, and lower but still significant VE in one season; 53% (95% confidence interval (CI): 33–67) in 2012/13, 55% (95% CI: 25–73) in 2013/14 and 18% (95% CI: 3–31) in 2014/15. In conclusion, seasonal influenza vaccination was associated with substantial reductions in influenza-specific hospitalisation, particularly in adults ≥ 65 years with underlying chronic conditions. With the use of population-based patient register data on influenza-specific outcomes it will be possible to obtain real-time estimates of seasonal influenza VE.

## Introduction

Annual vaccination against circulating influenza viruses remains the best strategy for preventing illness from influenza. A clear challenge, however, is that vaccine effectiveness (VE) varies from year to year [1]. These variations may be due to differences in antigenic match between the vaccine and the circulating strain, the immune status of those who are being vaccinated, or the time interval between vaccination and influenza outbreak.

Influenza outcome specificity is an important factor affecting VE estimates, since outcomes with low specificity will either underestimate or overestimate influenza VE [2,3]. Seasonal influenza VE uncertainty is an important reason for obtaining estimates for in-season vaccine effectiveness (IVE) as early as possible [2,4,5]. Such estimates may help guide the outbreak response, especially if there are signs of an antigenic mismatch that might require complementary public health measures.

There are controversies concerning the overall influenza VE, especially in elderly people, in most studies defined as adults ≥ 65 years of age [6,7]. Real-world evidence of vaccine effectiveness is therefore imperative for future influenza vaccine development and programme evaluation. The seasonal influenza vaccination programme in Stockholm offers vaccination at no out-of-pocket cost to individuals aged 65 years and older, pregnant women, and people of any age with certain underlying risk factors (chronic diseases of the heart, lungs, kidneys or liver, diabetes mellitus, neurological disease affecting the patient’s lung function, obesity with a body mass index of > 40, and immunosuppression caused by a disease or treatment). The actual benefit to these targeted groups is largely unknown and the aim of this study was therefore to develop methods for evaluating IVE and the overall seasonal vaccine effectiveness (VE) in all persons, irrespective of underlying risk factors, with medically attended influenza and pneumonia hospitalisations and primary care cases in Stockholm County, Sweden.

## Methods

### Study population and period

This study was based on four annual closed cohorts each comprising all individuals registered in Stockholm at the start of each season. The influenza season was defined as starting on 1 October and ending on 31 May the following year.

### Data sources

Data were collected using Stockholm County’s central database for healthcare utilisation, consultations and diagnoses, VAL. VAL has comprehensive inpatient, hospital outpatient, and primary care data and is used by the County Council to update the national patient register (PR) [8]. Multiple register linkages are possible due to unique personal identification numbers (PIN). Age and sex were retrieved from the primary care listing register in VAL. Immigration and death dates were not available in VAL, necessitating the design of a closed cohort for each season. We used the Stockholm Mosaic system as a proxy for living conditions and socioeconomic status [9]. The Mosaic system is based on eleven mutually exclusive categories (e.g. living in a low-income urban apartment block, multicultural suburb, affluent inner city, countryside, etc.) and involves 120 smaller urban agglomerations. Data on vaccine exposures were retrieved from the vaccination register, Vaccinera, which contains all data on seasonal influenza, pandemic influenza and pneumococcal vaccination of persons belonging to medical risk groups from the region, since 2009. Regional coverage in this database is assumed to be 100% as high-risk persons are vaccinated free of charge within the programme and registration is mandatory and required for reimbursements to the healthcare provider. Data on influenza status and comorbidities were obtained from the inpatient, hospital outpatient, and primary care databases.

### Case definition

Cases were defined as a clinical diagnosis of influenza during the season. International Classification of Diseases, 10th revision (ICD-10) codes J09 (influenza due to certain identified influenza viruses), J10 (influenza due to other identified influenza virus) and J11 (influenza due to unidentified influenza virus with pneumonia) were used to identify influenza diagnoses from inpatient, hospital outpatient, and primary care registers in VAL [10]. In a recent study VAL had over 99% coverage for inpatient care, 90% coverage for hospital outpatient care, and estimated 85% coverage for primary care [8]. National-level reporting estimates a validity of 85–95% for inpatient care, depending on the ICD-10 diagnosis [11]. Influenza cases were classified as inpatient cases if they came from the inpatient register and as outpatient cases if they came from the hospital outpatient or primary care registers. The inpatient register defined the case if an individual existed in multiple registers.

For the purpose of subanalysis, inpatient or outpatient non-influenza pneumonia, using ICD-10 codes J12-J18, was allowed.

Comorbidities were extracted from VAL using ICD-10 codes registered for a period of up to three years before the start of the respective season. ICD-10 codes for tumours (C00-D48), diabetes (E10–14) and circulatory (I00-I99) and non-acute respiratory illness (J40-J99) were extracted. 

### Vaccination status

Vaccination dates and seasonal vaccine type were derived from Vaccinera. Three different trivalent inactivated vaccines, Vaxigrip (Sanofi Pasteur MSD, Lyon, France), Fluarix (GSK, Brentford, United Kingdom), and Inflexal V (Crucell, Janssen Vaccines, Leiden, The Netherlands), were used during the seasons covered. No high-dose or adjuvanted vaccines were available in Sweden during the four seasons. Individuals with influenza infection before vaccination, or up to 13 days post-vaccination, were considered to be unvaccinated as were those who did not receive the seasonal vaccine. Those with influenza infection ≥ 14 days post vaccination were considered to be vaccinated. Pandemic influenza (Pandemrix, GSK) vaccination status from 2009/10 was included as a covariate as was pneumococcal vaccination (in the current season or previous seasons since 2009). Vaccination against seasonal influenza in the previous season was also included as a covariate.

### Influenza epidemiology

According to the Public Health Agency of Sweden, when compared with previous seasons, influenza activity was high during the most recent of the four seasons (2014/15), moderate during the 2011/12 and 2012/13 seasons, and low during the 2013/14 season [12] (Figure). Influenza A(H3N2) dominated in the 2011/12 and 2014/15 seasons, influenza A(H1N1)pdm09 dominated in 2013/14, whereas both these and influenza B viruses circulated in the 2012/13 season. There was also a significant amount of influenza B cases (approximately one-third of the cases) in 2014/15. In all four seasons influenza peaked during the second half of February.

**Figure:**
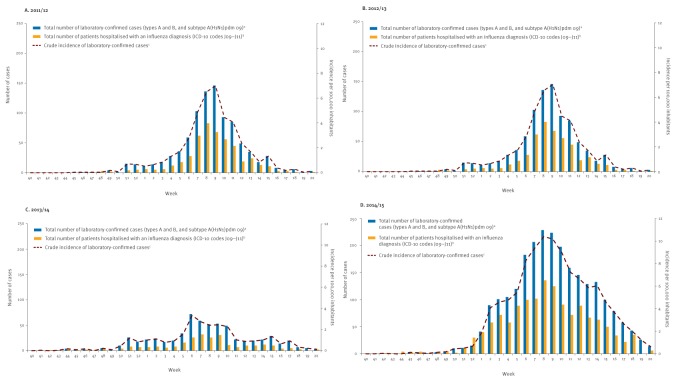
Number and incidence of laboratory-confirmed influenza cases, and number of patients hospitalised with influenza diagnosis in Stockholm County, influenza seasons 2011/12–2014/15

ICD: International Classification of Diseases.

Unadjusted incidence calculated by number of laboratory-confirmed cases per 100,000 inhabitants. Numbers reported by calendar week each season.

No data were available on number of laboratory-confirmed hospitalised influenza cases due to anonymous data in the central database for healthcare utilisation, making linkage impossible.

^a^ Data obtained from the Public Health Agency of Sweden

^b^ Data collected in Stockholm County’s central database for healthcare utilisation using ICD-10 codes J09-J11

^c^ (Number of laboratory-confirmed cases/number of inhabitants in Stockholm during season) × 100,000.

### Statistical analyses

Hazard rate ratios (HRR) comparing influenza inpatient and outpatient incidence among vaccinated and unvaccinated individuals were calculated using Cox regression analyses. Models were adjusted for age (grouped into 10-year intervals), sex, comorbidity status, socioeconomic status, pandemic vaccination, previous season influenza vaccination and pneumococcal vaccination. Stratified analysis of elderly people, aged 65 years or older, and individuals with underlying chronic illnesses was also performed, including age as a linear variable. Vaccination status was included as a time-varying exposure in the model, so individuals could contribute both vaccinated and unvaccinated risk time. In the final model comorbidity was adjusted for as a dichotomous variable as yes or no. The overall seasonal influenza vaccine effectiveness (VE) was calculated as (1 − HRR) x 100%. Both HRR and VE were reported with 95% confidence intervals (CI).

Additional regression analyses modelled VE on inpatient and outpatient pneumonia (ICD-10 J12-J18), adjusting for age (grouped into 10-year intervals), sex, comorbidity status, pandemic vaccination, previous season influenza vaccination and pneumococcal vaccination.

Regression analyses for the pre-influenza periods, 1 June to 30 September of the four seasons under investigation were performed to assess whether there was a healthy-vaccinee bias present in the cohort. Previous studies have reported on such a bias, which would augment VE estimates [13,14]. Pre-season analyses modelling influenza among those vaccinated later during the season were adjusted for age, sex and comorbidity status.

Data management and analyses were carried out using SAS Enterprise software (SAS Institute Inc., Cary, NC).

### Ethical consideration

This analysis was part of ongoing programme evaluations required at the Department of Communicable Disease Control and Prevention, Stockholm County Council, Stockholm, Sweden. As this evaluation was a requisite part of Stockholm County Council work processes, it falls outside the mandate for the Regional Ethics committee. PINs have been anonymised in VAL and no data making individual identification possible is retained.

## Results

In total, 2–2.2 million individuals were included per season in the study (Tables 1A and 1B). A slightly higher proportion of women were vaccinated compared to men. (Tables 1A and 1B). The number of patients with a clinical diagnosis of influenza was highest in 2011/12 and in 2014/15, seasons dominated by influenza A(H3N2), but the need for hospital treatment was about three times higher in 2014/15 than in 2011/12 (Table 2). The number of people hospitalised with a diagnosis of influenza during the influenza seasons followed the curve of laboratory-confirmed cases in the county (Figure).

**Table 1A:** Baseline characteristics of the cohorts in influenza analysis, Stockholm County, influenza seasons 2011/12 and 2012/13

Characteristic	Influenza season 2011/12			Influenza season 2012/13		
	Total	Vaccinated^a^	Unvaccinated	Total	Vaccinated^a^	Unvaccinated
	n	n (%)	n (%)	n (%)	n (%)	n (%)
Cohort total	2,089,047	205,415 (9.8)	1,883,612 (90.2)	2,121,469	185,646 (8.8)	1 935,823 (91.2)
Sex						
Male	1,034,494	87,659 (8.5)	946,835 (91.5)	1,051,818 (49.6)	79,920 (7.6)	971,898 (92.4)
Female	1,054,553	117,756 (11.2)	936,797 (88.8)	1,069,651 (50.4)	105,726 (9.9)	963,925 (90.1)
Age group in years						
< 10	270	388 (0.1)	270,435 (99.9)	276,358 (13.0)	273 (0.1)	276,085 (99.9)
10–19	232	540 (0.2)	231,971 (99.8)	231,869 (10.9)	388 (0.2)	231,481 (99.8)
20–29	283,977	1,373 (0.5)	282,604 (99.5)	291,993 (13.8)	1,014 (0.4)	290,979 (99.6)
30–39	320,932	3,219 (1.0)	317,713 (99.0)	322,867 (15.2)	2,437(0.8)	320,430 (99.2)
40–49	307,966	4,457 (1.4)	303,509 (98.6)	313,605 (14.8)	3,499 (1.1)	310,106 (98.9)
50–59	241,944	8,340 (3.4)	233,604 (96.6)	246,848 (11.6)	6,916 (2.8)	239,932 (97.2)
60–69	223,956	60,580 (27.0)	163,376 (73.0)	224,713 (10.6)	52,719 (23.5)	171,994 (76.5)
70–79	121,415	73,510 (60.5)	47,905 (39.5)	127,570 (6.0)	70,014 (54.9)	57,556 (45.1)
≥ 80	85,523	53,008 (62.0)	32,515 (38.0)	85,646 (4.0)	48,386 (56.5)	37,260 (43.5)
Mosaic income/education categories						
Highest income and education	945,893	94,506 (10.0)	851,387 (90.0)	971,845	85,992 (8.8)	885,853 (91.2)
Middle income and education	360,980	36,871 (10.2)	324,109 (89.8)	372,925	33,095 (8.9)	339,830 (91.1)
Lowest income and education	744,905	73,067 (9.8)	671,838 (90.2)	761,746	66,032 (8.7)	695,714 (91.3)
Missing	37,269	1,450 (3.9)	35,819 (96.1)	14,653	536 (3.7)	14,117 (96.3)
Comorbidity						
Yes	586,470	148,196 (25.3)	438,274 (74.7)	613,183 (28.9)	138,020 (22.5)	475,163 (77.5)
No	1,502,577	57,219 (3.8)	1,445,358 (96.2)	1,508,286 (71.1)	47,626 (3.2)	1,460,660 (96.8)
Previous seasonal vaccination^b^						
Yes	203,736	162,379 (79.7)	41,357 (20.3)	198,361 (9.4)	151,359 (76.3)	47,002 (23.7)
No	1,885,311	43,036 (2.3)	1,842,275 (97.7)	1,923,108 (90.7)	34,287 (1.8)	1,888,821 (98.2)
Pneumococcal vaccination^c^						
Yes	33,374	28,232 (84.6)	5,142 (15.4)	39,502 (1.9)	30,891 (78.2)	8,611 (21.8)
No	2,055,673	177,183 (8.6)	1,878,490 (91.4)	2,081,967 (98.1)	154,755 (7.4)	1,927,212 (92.6)
Pandemrix vaccination^d^						
Yes	1,064,132	163,246 (15.3)	861,669 (84.7)	1,007,546 (47.5)	148,338 (14.7)	859,208 (85.3)
No	1,024,915	42,169 (4.1)	1,021,963 (95.9)	1 113 923 (52.5)	37,308 (3.4)	1,076,615 (96.6)

Influenza season defined as 1 October to 31 May in the following year.

^a^ During the 2011/12 influenza season, 99.5% of seasonal influenza vaccinations were carried out using Vaxigrip. During 2012/13, 99.4% of seasonal influenza vaccinations were carried out using Fluarix.

^b^ Vaccinated against seasonal influenza during the previous season.

^c^ Vaccinated against Streptococcus pneumoniae during time period from 2009 to the season under investigation.

^d^ Vaccinated against influenza A(H1N1)pdm09 during the 2009 pandemic.

**Table 1B:** Baseline characteristics of cohorts, Stockholm County, influenza seasons 2013/14 and 2014/15

Characteristic	Influenza season 2013/14			Influenza season 2014/15		
	Total	Vaccinated^a^	Unvaccinated	Total	Vaccinated^a^	Unvaccinated
	n	n (%)	n (%)	n (%)	n (%)	n (%)
Cohort total	2,171,207	199,707 (9.2)	1,971,500 (90.8)	2,207,172	205,709 (9.3)	2,001,463 (90.7)
Sex						
Male	1,077,657	84,692 (7.9)	992,965 (92.1)	1,096,957 (49.7)	88,091 (8.0)	1,008,866 (92.0)
Female	1,093,550	115,015 (10.5)	978,535 (89.5)	1,110,215 (50.3)	117,618 (10.6)	992,597 (89.4)
Age group in years						
< 10	283,541	488 (0.2)	283,053 (99.8)	287,422 (13.0)	495 (0.2)	286,927 (99.8)
10–19	234,837	521 (0.2)	234,316 (99.8)	236,884 (10.7)	606 (0.3)	236,278 (99.7)
20–29	305,611	1,892 (0.6)	303,719 (99.4)	311,773 (14.1)	2,257 (0.7)	309,516 (99.3)
30–39	327,012	4,715 (1.4)	322,297 (98.6)	330,199 (15.0)	5,343 (1.6)	324,856 (98.4)
40–49	319,407	4,371 (1.4)	315,036 (98.6)	323,168 (14.6)	5,116 (1.6)	318,052 (98.4)
50–59	254,154	7,906 (3.1)	246,248 (96.9)	263,216 (11.9)	9,094 (3.5)	254,122 (96.5)
60–69	224,687	54,003 (24.0)	170,684 (76.0)	222,631 (10.1)	52,957 (23.8)	169,674 (76.2)
70–79	136,323	76,112 (55.8)	60,211 (44.2)	146,285 (6.6)	79,824 (54.6)	66,461 (45.4)
≥ 80	85,635	49,699 (58.0)	35,936 (42.0)	85,594 (3.9)	50,017 (58.4)	35,577 (41.6)
Mosaic income/education						
Highest income and education	990,078	93,330 (9.4)	869,748 (90.6)	1,001,695	97,153 (9.7)	904,542 (90.3)
Middle income and education	381,870	36,128(9.5)	345,742 (90.5)	389,999	37,076 (9.5)	352,923 (90.5)
Lowest income and education	776,802	69,729 (9.0)	707,073 (91.0)	786,842	70,113 (8.8)	716,729 (91.2)
Missing	22,457	890 (4.0)	21,567 (96.0)	28,636	1,367 (4.8)	27,269 (95.2)
Comorbidity						
Yes	635,947	147,899 (23.3)	488,048 (76.7)	653,248 (29.6)	15,187 (23.2)	501,421 (76.8)
No	1,535,260	51,808 (3.4)	1,483,452 (96.6)	1,553,924 (70.4)	53,882 (3.5)	1,500,042 (96.5)
Previous seasonal vaccination^b^						
Yes	179,658	149,881 (83.4)	29,777 (16.6)	193,432 (8.8)	153,515 (79.4)	39,917 (20.6)
No	1,991,549	49,826 (2.5)	1,941,723 (97.5)	2,013,740 (91.2)	52,194 (2.6)	1,961,546 (97.4)
Pneumococcal vaccination^c^						
Yes	48,009	38,801 (80.8)	9,208 (19.2)	55,929 (2.5)	43,833 (78.4)	12,096 (21.6)
No	2,123,198	160,906 (7.6)	1,962,292 (92.4)	2,151,243 (97.5)	161,876 (7.5)	1,989,367 (92.5)
Pandemrix vaccination^d^						
Yes	995,193	156,389 (15.7)	838,804 (84.3)	981,065 (44.5)	157,771 (16.1)	823,294 (83.9)
No	1,176,014	43,382 (3.7)	1,132,632 (96.3)	1 226 107 (55.5)	47,938 (3.9)	1,178,169 (96.1)

Influenza season defined as 1 October to 31 May in the following year.

^a^ During 2013/14 and 2014/15, 99.4% of seasonal influenza vaccination were carried out using Fluarix.

^b^ Vaccinated against seasonal influenza during the previous season.

^c^ Vaccinated against *Streptococcus pneumoniae* during time period from 2009 to the current season under investigation.

^d^ Vaccinated against influenza A(H1N1) during the 2009 pandemic.

**Table 2:** Hazard ratios with 95% confidence intervals and vaccine effectiveness estimates for seasonal influenza vaccination on influenza outcome including inpatient and outpatient cases, Stockholm County, influenza seasons 2011/12–2014/15

Category	Total number	All cases			Outpatient			Inpatient		
		Cases	HR (95% CI)	VE	Cases	HR (95% CI)	VE	Cases	HR (95% CI)	VE
2011/12										
All										
Unvaccinated	1,883,612	5,109	Ref	NA	4,793	Ref	NA	316	Ref	NA
Vaccinated	205,415	374	0.81 (0.69–0.94)	19% (6–31)	210	0.69 (0.57–0.84)	31% (14–43)	164	1.07 (0.79–1.46)	0
Age ≥ 65 years										
Unvaccinated	140,143	263	Ref	NA	161	Ref	NA	102	Ref	NA
Vaccinated	176,622	299	0.90 (0.72–1.12)	10% (0–28)	149	0.86 (0.64–1.17)	14% (0–36)	150	0.94 (0.68–1.31)	6% (0–32)
2012/13										
All										
Unvaccinated	1,935,823	2,471	Ref	NA	1,885	Ref	NA	586	Ref	NA
Vaccinated	185,646	139	0.60 (0.48–0.77)	40% (23–52)	48	0.55 (0.37–0.81)	45% (19–63)	91	0.53 (0.39–0.73)	47% (27–61)
Age ≥ 65 years										
Unvaccinated	163,988	202	Ref	NA	55	Ref	NA	147	Ref	NA
Vaccinated	162,678	106	0.51 (0.38–0.69)	49% (31–62)	31	0.62 (0.35–1.10)	38% (0–65)	75	0.48 (0.34–0.69)	52% (31–66)
2013/14										
All										
Unvaccinated	1,971,500	2,076	Ref	NA	1,850	Ref	NA	226	Ref	NA
Vaccinated	199,707	105	0.63 (0.48–0.83)	37% (17–52)	57	0.58 (0.41–0.83)	42% (17–59)	48	0.70 (0.44–1.11)	30% (0–56)
Age ≥ 65 years										
Unvaccinated	166,024	129	Ref	NA	58	Ref	NA	71	Ref	NA
Vaccinated	170,752	74	0.54 (0.37–0.79)	46% (21–56)	33	0.59 (0.33–1.05)	41% (0–67)	41	0.51 (0.31–0.83)	49% (17–69)
2014/15										
All										
Unvaccinated	2,001,463	4829	Ref	NA	3,980	Ref	NA	849	Ref	NA
Vaccinated	205,709	829	0.85 (0.76–0.95)	15% (5–24)	298	0.83 (0.70–0.98)	17% (2–30)	531	0.84 (0.72–0.99)	16% (1–28)
Age ≥ 65 years										
Unvaccinated	172,245	697	Ref	NA	212	Ref	NA	485	Ref	NA
Vaccinated	173,075	705	0.82 (0.71–0.93)	18% (7-29)	204	0.89 (0.69–1.15)	11% (0–31)	501	0.79 (0.68–0.93)	21% (7-32)

CI: confidence interval; HR: hazard ratio; NA: not applicable; Ref: reference value; VE: vaccine effectiveness.

International Classification of Diseases, 10th revision codes J09-J11 were used to identify influenza diagnoses [10].

Vaccine effectiveness calculated (1 – HR × 100).

Hazard ratios derived from Cox proportional hazards regression model; adjusted for sex, age (age groups 10-year intervals), comorbidity status, socioeconomic status, previous seasonal vaccination, pneumococcal vaccination and Pandemrix vaccination. As complete case analysis was used, the number of cases decreased due to missing in socioeconomic status.

In 2011/12, more than 99% of all those vaccinated received Vaxigrip, while in the remaining seasons more than 99% were vaccinated with Flurarix. Almost 30% of the individuals included in the analysis had a documented comorbidity and of these ca 25% were vaccinated. There were no differences in vaccination rates among those with high or low socioeconomic status (Tables 1A and 1B).

For the 2011/12 season, overall VE for inpatient and outpatient care was 19% (95% CI: 6–31), driven primarily by outpatient effects in those younger than 65 years of age (Table 2). For the 2012/13 season, overall VE was higher, 40% (95% CI: 23–52), with stronger VE seen among inpatients, particularly those 65 years of age or older (VE: 52%; 95% CI: 31–66). For the 2013/14 season, overall VE was 37% (95% CI: 17–52), with elderly inpatient care driving the effects (VE: 49%; 95% CI: 17–69 for those 65 years or older). In 2014/15, the study season with the highest burden of hospital treatment of influenza, the VE was again lower and the vaccine effect was strongest for those 65 years, or older, 18% (95 CI: 7–29) overall and 21% (95% CI: 7–32) for inpatient care.

For the two seasons with moderately high VEs, inpatient VE for patients with comorbidities was similar to that of the whole population (Table 3). Stratified analyses on comorbidity showed 48–55% effectiveness against inpatient care in the seasons 2012/13 and 2013/14 for those with underlying chronic illness, both overall and among those 65 years of age or older. VE in outpatient care was not as strongly affected by comorbidity status.

**Table 3:** Stratified analyses presenting hazard ratios with 95% confidence intervals and vaccine effectiveness estimates for seasonal influenza vaccination on influenza outcome among individuals with comorbidity, Stockholm County, influenza seasons 2011/12–2014/15

Category	Total number	All cases among those with comorbidity			Outpatient among those with comorbidity			Inpatient among those with comorbidity		
		Cases	HR (95% CI)	VE	Cases	HR (95% CI)	VE	Cases	HR (95% CI)	VE
2011/12										
All										
Unvaccinated	438,274	1,624	Ref	NA	1,424	Ref	NA	200	Ref	NA
Vaccinated	148,196	307	0.79 (0.66–0.95)	21% (5–34)	164	0.71 (0.57–0.90)	14% (0.36)	143	0.90 (0.65–1.24)	10% (0–35)
Age ≥ 65 years										
Unvaccinated	8,205	193	Ref	NA	103	Ref	NA	90	Ref	NA
Vaccinated	131,456	249	0.85 (0.66–1.09)	15% (0–34)	117	0.87 (0.61–1.23)	13% (0–39)	132	0.83 (0.60–1.18)	17% (0–40)
2012/13										
All										
Unvaccinated	475,163	949	Ref	NA	607	Ref	NA	342	Ref	NA
Vaccinated	138,020	117	0.54 (0.41–0.70)	46% (30–59)	36	0.56 (0.35–0.89)	44% (11–65)	81	0.50 (0.36–0.69)	50% (31-64)
Age ≥ 65 years										
Unvaccinated	106,110	180	Ref	NA	47	Ref	NA	133	Ref	NA
Vaccinated	123,472	94	0.47 (0.34–0.64)	53% (37–66)	24	0.51 (0.27–0.95)	49% (5–73)	70	0.47 (0.33–0.67)	53% (33–67)
2013/14										
All										
Unvaccinated	488,048	745	Ref	NA	604	Ref	NA	141	Ref	NA
Vaccinated	147,899	93	0.70 (0.51–0.95)	30% (5-49)	51	0.81 (0.54–1.21)	19% (0–46)	42	0.52 (0.32–0.84)	48% (16–68)
Age ≥ 65 years										
Unvaccinated	108,496	104	Ref	Ref	37	Ref	Ref	67	Ref	Ref
Vaccinated	130,592	67	0.55 (0.37–0.82)	45% (18-63)	30	0.78 (0.41–1.48)	22% (0–59)	37	0.45 (0.27–0.75)	55% (25–73)
2014/15										
All										
Unvaccinated	501,421	2,002	Ref	Ref	1,391	Ref	Ref	611	Ref	Ref
Vaccinated	151,827	731	0.85 (0.75–0.97)	15% (3-25)	237	0.84 (0.68–1.03)	16% (0–32)	494	0.85 (0.72–1.00)	15% (0–28)
Age ≥ 65 years										
Unvaccinated	113,444	591	Ref	NA	164	Ref	NA	427	Ref	NA
Vaccinated	133,226	639	0.82 (0.71–0.94)	18% (6-29)	169	0.84 (0.63–1.11)	16% (0–37)	470	0.82 (0.69–0.97)	18% (3-31)

CI: confidence interval; HR: hazard ratios; NA: not applicable; Ref: reference value; VE: vaccine effectiveness

International Classification of Diseases, 10th revision codes J09–J11 were used to identify influenza diagnoses. [10]

^a^ Vaccine effectiveness, calculated (1 − HR × 100).

^b^ Hazard ratios derived from Cox proportional hazards regression model; adjusted for sex, age (age groups 10 years intervals), socioeconomic status, previous seasonal vaccination, pneumococcal vaccination and Pandemrix vaccination. As complete case analysis was used, the number of cases decreased due to missing in socioeconomic status.

Stratified analyses on previous season influenza vaccination among those 65 years of age or older, showed no clear effects, either protective or negative, against the risk of being hospitalised with a diagnosis of influenza in the current season (data not shown).

The pre-influenza season analyses, 1 June to 30 September, were all statistically insignificant, with HRs of 1.71 (95% CI: 0.80–3.66), 0.87 (95% CI: 0.29–2.56), 1.09 (95% CI: 0.45–2.65), and 0.83 (95% CI: 0.34–2.01), respectively, indicating that vaccination was not associated with either a decreased or increased risk of receiving a diagnosis of influenza in any of these four pre-influenza season periods.

VE for inpatient non-influenza pneumonia in persons aged 65 years or older ranged from 11% to 18% during the four seasons. No effectiveness could be demonstrated against non-hospitalised pneumonia (Table 4).

**Table 4:** Hazard ratios with 95% confidence intervals and vaccine effectiveness estimates for seasonal influenza vaccination on pneumonia outcome including inpatient and outpatient cases, Stockholm County, influenza seasons 2011/12–2014/15

Category	Total number	All cases			Outpatient			Inpatient		
		Cases	HR^a^ (95% CI)	VE^b^	Cases	HR^a^ (95% CI)	VE^b^	Cases	HR^a^ (95% CI)	VE^b^
2011/12										
All										
Unvaccinated	1,884,818	20,088	Ref	NA	15,123	Ref	NA	4,965	Ref	NA
Vaccinated	204,229	4,849	1.15 (1.09–1.20)	0	2,267	1.28 (1.20–1.37)	0	2,582	0.97 (0.90–1.07)	3% (0–10)
Age ≥ 65 years										
Unvaccinated	141,097	4,735	Ref	NA	1,878	Ref	NA	2,857	Ref	NA
Vaccinated	175,668	4,333	0.93 (0.88–0.99)	7% (1–12)	1,946	1.14 (1.04–1.24)	0	2,387	0.82 (0.76–0.88)	18% (12–24)
2012/13										
All										
Unvaccinated	1,936,790	10,224	Ref	NA	8,013	Ref	NA	2,211	Ref	NA
Vaccinated	184,679	3,606	1.02 (0.96–1.07)	0	1,518	1.05 (0.97–1.13)	0	2,088	0.97 (0.90–1.04)	3% (0–10)
Age ≥ 65 years										
Unvaccinated	164,807	4,697	Ref	NA	1,787	Ref	NA	2,910	Ref	NA
Vaccinated	161,859	3,250	0.95 (0.89–1.00)	5% (0–11)	1,319	1.05 (0.96–1.16)	0	1,931	0.89 (0.83–0.96)	11% (4–17)
2013/14										
All										
Unvaccinated	1,972,363	12,718	Ref	NA	8,527	Ref	NA	4,191	Ref	NA
Vaccinated	198,844	3,737	1.05 (1.00–1.11)	0	1,700	1.20 (1.11–1.29)	0	2,037	0.95 (0.88–1.03)	5% (0–12)
Age ≥ 65 years										
Unvaccinated	166,773	4,247	Ref	NA	1,633	Ref	NA	2,614	Ref	NA
Vaccinated	170,003	3,349	1.00 (0.94–1.06)	0	1,455	1.20 (1.09–1.32)	0	1,894	0.89 (0.82–0.96)	11% (4-18)
2014/15										
All										
Unvaccinated	2,002,587	16,155	Ref	NA	11,336	Ref	NA	4,819	Ref	NA
Vaccinated	204 585	4 636	1.08 (1.03–1.13)	0	2,207	1.17 (1.09–1.25)	0	2,429	0.97 (0.91–1.04)	3% (0–9)
Age ≥ 65 years										
Unvaccinated	166 773	5 264	Ref	NA	2,221	Ref	NA	3,043	Ref	NA
Vaccinated	173 170	4 146	0.98 (0.93–1.03)	2% (0–7)	1,905	1.12 (1.03–1.22)	0	2,241	0.89 (0.82–0.95)	11% (5–18)

CI: confidence interval; HR: hazard ratio; NA: not applicable; Ref: reference value; VE: vaccine effectiveness.

International Classification of Diseases, 10th revision codes J12-J18 were used to identify non-influenza pneumonia diagnoses [10].

^a^ Hazard ratios derived from Cox proportional hazards regression model; confidence interval; adjusted for sex, age (age groups 10 years intervals), comorbidity status, socioeconomic status, previous seasonal vaccination, pneumococcal vaccination and Pandemrix vaccination. As complete case analysis was used, the number of cases decreased due to missing in socioeconomic status.

^b^ Vaccine effectiveness calculated (1 – HR × 100).

## Discussion

In this study we used influenza and pneumonia diagnosis codes linked with vaccination status from the entire population of a large metropolitan area to evaluate seasonal influenza vaccine effectiveness on inpatient hospitalisations and primary care visits. Our results thus provide important real-world vaccination programme effects in individuals of varying ages and health statuses. Vaccine effects were moderately good both in adults <65 years of age and in elderly people (≥ 65 years of age), including those with comorbidities, during two of the four seasons. Small but significant VE against non-influenza pneumonias was found in persons 65 years or older in all four seasons. However, since the proportion of pneumonia caused by influenza in most studies is less than 20%, a VE of 11–18% for pneumonia hospitalisation in persons aged 65 years or older, of whom about half were vaccinated, could indicate a VE for influenza-related pneumonia as high as 50–75% [3].

Seasonal influenza programme vaccination is typically recommended to prevent severe outcomes in highly vulnerable groups. What constitutes optimal outcome measures for seasonal influenza VE is debatable, however. Commonly used outcome measures are influenza-like-illness (ILI), acute respiratory infection (ARI), or hospitalisation for influenza or pneumonia [6,15,16]. Effectiveness against laboratory-confirmed influenza vaccine type is the most specific outcome measure, although often available for relatively limited populations, such as healthy adults, and as such not fully generalisable to populations targeted for influenza programmes [2,4].

The four pre-influenza season period analyses did not show any difference in the risk of receiving a clinical diagnosis of influenza in vaccinated vs non-vaccinated persons, indicating that there was no healthy-vaccinee bias in the current study. This is in contrast to most studies, including an earlier study from Stockholm [13,14,17,18]. The former Stockholm study was performed in 1998–2001 when the yearly seasonal influenza vaccination campaigns were new and included only adults aged 65 years or older. Vaccines were not offered free of charge as they are today, which may also explain the healthy-vaccinee bias found in that study [14]. In addition, during the last few years, Stockholm’s influenza vaccine campaign has been developed specifically to target the chronically ill, irrespective of age.

Randomised control trials (RCTs) measuring influenza VE among elderly people are rare and the only one of high quality showed a 50% effect against serologically confirmed influenza [19]. Pooled observational studies have shown nominal effects among the elderly in nursing homes (ILI VE 23%; hospitalisation for pneumonia VE 45%), but non-significant effects on elderly people living in the community in terms of ILI or influenza [6]. Overall, observational VE estimates range from 25% to 60% in protecting against hospitalisation for influenza or pneumonia among the elderly [6,16,20]. Observational studies are often not able to account for specific effects among the chronically ill, which is a major limitation [16]. When treatment choice, or in this case vaccination status, is driven by an individual’s disease status, it is referred to as confounding by indication and is another type of selection bias. The influenza vaccination programme promotes this population selection bias by targeting those with underlying comorbidities. A major strength in our study is that these effect results have accounted for this major bias by linking with patient records and adjusting for comorbidity status. Other strengths were that we adjusted for potential differences stemming from socioeconomic status and controlled for residual effects in seasonal VE estimates due to previous seasonal vaccinations [21,22], pandemic influenza and pneumococcal vaccinations.

The European network Influenza - Monitoring Vaccine Effectiveness (I-MOVE) has monitored VE in a number of countries since 2008 by observational studies using the ‘test-negative’ or ‘screening’ designs [1]. Our results among persons with comorbidity showing a very low VE in 2011/12, but a moderately good VE around 50% for prevention of hospitalisation for influenza among persons aged 65 years or older in 2012/13 and 2013/14, are in accordance with those presented by I-MOVE. They found a very low VE during the 2011/12 season, from 43% during the early part of the season down to less than 10% in risk groups when the whole season was analysed [23,24]. The reason for this low VE late in the 2011/12 season may have been a waning vaccine effect in older persons, since the peak came late in the season, or an antigenic drift [24]. During the 2012/13 season, when all three influenza types circulated, I-MOVE reported a moderately high VE in Europe (43–63% depending on influenza type), and also in 2013/14 with a VE for the dominating influenza A(H1N1)pdm09 of 48% [23,25]. Reports from the 2014/15 season from North America and Europe are in accordance with our findings that VE was lower than during the two preceding seasons [26-28]. A possible reason for this lower VE is that circulation of newly emerged A(H3N2) clades 3C.3a and 3C.2a viruses, to which antibodies in humans to the A/Texas/50/2012 antigens contained in the seasonal vaccine, reacted less well [28,29].

Effects among adults under 65 years of age, particularly healthy individuals, should theoretically be higher than in elderly people, as they have a better immune response to vaccination. In contrast, VE among healthy adults below 65 years in our study was similar to, or lower than among the elderly. A possible reason for this finding is a potential misclassification of exposure, since entering influenza vaccination of healthy adults below 65 years in the vaccination register is not requisite, as Stockholm neither recommends nor subsidises influenza vaccinations for these individuals. If healthy individuals aged under 65 years obtain vaccinations via mobile clinics at their workplace or via a healthcare provider, they may not be entered in the vaccination register. As such, some may be inappropriately classified as unvaccinated in our study, and hence weaken the effect measures of VE. In contrast, persons belonging to risk groups according to the programme will most likely have been registered in the vaccination register, since they are offered the vaccine free of charge and have easy access to caregivers included in the programme. In addition, caregivers are reimbursed only when they adhere to the reporting requirements.

Although we did not see any evidence of a healthy-vaccinee bias in pre-season analyses, the power of this analysis was low since the few cases with influenza diagnoses off-season resulted in wide confidence intervals. Another limitation is that VAL experienced a technical problem while merging primary care data for 2013, and thus it appears as if there are a reduced number of primary care cases for this year. This technical problem is non-differential and, if anything, would generate diluted VEs. Inpatient care is complete and not affected by these technicalities. We could not control for the severity of comorbidity or the severity of the acute disease in order to identify patients in need of intensive care treatment, nor could we analyse mortality outcomes, since these data are not included in the County’s surveillance. Negative controls were not included in these analyses, although pneumonia was included as a subanalysis, and while significant VE was found, it was very low because of the diluting effect of such a non-specific diagnosis.

Our study found robust VE against influenza hospitalisation, a proxy for severe disease. This VE was most substantial among adults and the elderly having underlying chronic conditions. Therefore, we believe that public health officials should focus resources also on attaining high coverage in people with underlying diseases, irrespective of age, in addition to the WHO/EU goal of a 75% for coverage among all people 65 years of age or older [30].

The need for additional effectiveness studies for the influenza vaccine with non-specific outcomes such as pneumonia or influenza-like illness has been questioned since the potential for overestimation or underestimation of vaccine effectiveness is too great [3]. Although the influenza diagnoses were not laboratory-confirmed, our study demonstrates that comprehensive population-based patient register data on influenza-specific outcomes, which allow for adjustments of multiple confounders and assessments of potential biases, can and should be used for routine estimates of seasonal influenza IVE and VE. The VEs in our study were in accordance with those from European multicentre studies using the much more laborious test-negative design [25,31,32]. International sentinel surveillance efforts remain vital to gauge circulating types, but are not needed to accurately assess VE across broad populations. In addition, large and expensive RCTs to estimate effects of seasonal influenza vaccines are neither fiscally nor ethically justifiable in the era of reliable electronic medical record data.

Since the beginning of 2016 we have had a regular weekly linkage between Stockholm’s central database for healthcare diagnoses, VAL, and the vaccine register [33]. These real-time data showed that the 55–68% IVE seen in persons aged 65 years or older during January and February, when A(H1N1)pdm09 dominated, declined when influenza B (Victoria) took over and was only 43–44% from the end of March, an observation which lead us to take action and recommend that doctors prescribe early antiviral therapy for ILI in this patient group.

In conclusion, results from this population-based evaluation of multiple vaccine seasons show substantial protective VE against being hospitalised with a diagnosis of influenza among elderly and chronically ill persons in all age groups during two of four seasons and lower, but still significant, VE in another. Programmes that target these vulnerable populations can anticipate ca 50% reductions in influenza-specific inpatient care, in seasons with a good antigenic match. We also demonstrate that the use of population-based patient register data on influenza-specific outcomes enables valuable real-time estimates of seasonal influenza vaccine effectiveness.
